# Fear of Childbirth Among Pregnant People Facing Anti-Fat Bias

**DOI:** 10.3390/ijerph21121657

**Published:** 2024-12-11

**Authors:** Lee Roosevelt, Sarah Maguire, Akshay Sharma, Ruth Zielinski

**Affiliations:** 1 School of Nursing, University of Michigan, Ann Arbor, MI 48109, USA; akshaydr@med.umich.edu (A.S.); ruthcnm@umich.edu (R.Z.); 2 Midwifery Service, University of Michigan Health, Ann Arbor, MI 48109, USA; maguires@umich.edu

**Keywords:** fear of childbirth, anti-fat bias, pregnancy outcomes

## Abstract

Explicit and implicit anti-fat biases are widespread among healthcare providers, leading to significant negative consequences for pregnant people, including poorer health outcomes. Fear of childbirth (FOC) can affect the length of labor, increase the risk of cesarean delivery, and negatively influence a new parent’s perception of infant bonding. This study investigated the impact of perceived anti-fat bias on FOC among pregnant people. Data were gathered from 329 pregnant people recruited from three large academic prenatal centers in the United States and via social media. Participants completed a survey that included validated instruments measuring perceptions of anti-fat bias and FOC. Participants perceiving anti-fat bias reported higher FOC. Black participants perceiving anti-fat bias reported higher FOC. These findings suggest that perceived anti-fat bias from providers is associated with FOC for pregnant people, particularly those who identify as Black. Interventions to educate providers on these important concepts could help improve pregnant people’s experience within the healthcare system.

## 1. Introduction

The concept of fear of childbirth (FOC) has been used in the literature as a broad label for a wide variety of anxieties and fears that people experience in relation to pregnancy and childbirth [1]. In some of the first studies of FOC, the prevalence for pregnant people in Scandinavia was reported as 20%, with approximately 5% to 10% of people experiencing intense fear [2]. FOC is a complex phenomenon that represents the convergence of various feelings of anxiety during pregnancy into a single, socially and psychologically understandable entity [3], but has significant implications for negative health outcomes, including longer labors, higher rates of cesarean births, and more overall interventions [1,4,5]. Pregnant people experience fear of complications, fear of something happening to themselves or their baby, fear of pain, and fear of not having enough strength to give birth. However, many people also express fear of how their care providers will treat them and how larger pervasive hierarchies and structural aspects of the maternity care system, such as anti-fat bias, will affect the care they receive during birth [3].

Anti-fat bias is the set of destructive attitudes, opinions, and judgments made toward an individual due to their weight, particularly for those labeled as “overweight” or “obese” [6]. The classification of “obesity” (body mass index of 30 or greater) as an epidemic in public health and as a disease by the American Medical Association (AMA) resulted in obesity being labeled as both a social problem and a pathological condition needing remediation [7]. For the purposes of this paper, we have chosen to use the language utilized by the National Association to Advance Fat Acceptance (NAAFA). NAAFA uses the word “fat” to describe larger-bodied people that the medical model may refer to as “overweight” or “obese” [8]. The definition of “fat” as per the NAAFA’s constitution is: “characterizing an individual whose body weight exceeds that assigned to him or her by commonly accepted medical standards or who is commonly regarded as having excess weight” [8]. We concur with social scientists who highlight how the words “overweight” and “obese” can cause harm to fat people under the guise of objectivity [9].

The concern for increased risk status associated with fat bodies becomes magnified in pregnancy. This concern is widely acknowledged by practitioners and researchers on the basis of a robust body of literature documenting that a fat person is at increased risk for gestational diabetes, hypertensive disorders of pregnancy, greater total pregnancy weight gain, postpartum hemorrhage, cesarean birth, macrosomia, a low 5 min Apgar score, admission to the NICU, and early neonatal death [10,11,12,13]. Although the goal of identifying these issues has been to guide healthcare provider counseling and care recommendations [14] with the intention of improving health and reducing disparities, it has unfortunately had the harmful impact of reinforcing anti-fat bias [15,16].

Fat pregnant people often face significant discrimination in healthcare settings. Research with a range of healthcare providers, including physicians, nurses, midwives, occupational therapists, podiatrists, and psychologists, has demonstrated pervasive implicit and explicit anti-fat biases [16,17,18,19,20,21,22]. According to some patient reports, midwives are less likely than obstetricians and other healthcare providers to exhibit anti-fat bias [23,24]; however, another study found implicit bias was held by >70% of midwives [25]. Pregnant people have reported experiences with providers being insensitive, inappropriate, shaming, or ill-at-ease when discussing weight, as well as a lack of appropriately sized equipment and furniture, among other concerns [19,26,27,28]. The repercussions of anti-fat bias in health care are extensive. People experiencing provider anti-fat bias are less likely to disclose personal information due to mistrust or fear and more likely to miss follow-up care visits [15,29]. They are also more likely to experience greater gestational weight gain, postpartum weight retention, postpartum depression, maladaptive eating patterns, stress, depression, anxiety, and negative pregnancy outcomes [20,22,30].

Despite a growing body of research examining both FOC and anti-fat bias, there is a notable lack of studies examining the relationship between these two areas. Our aim is to shed light on the intersection of FOC and anti-fat bias that pregnant people may encounter to develop a more comprehensive understanding of the factors affecting the childbirth experience.

## 2. Methods

### 2.1. Positionality

The authors share their experiences and perspectives to provide clarity on how the research was approached. LR, SM, AS, and RZ are all health researchers with a specific focus on studying the healthcare experiences of underrepresented communities. LR, SM, and RZ are also practicing nurse-midwives who have given birth. AS is a physician and epidemiologist. LR, SM, and RZ all identify as white cisgender women. AS identifies as a South Asian cisgender man. LR and RZ both identify as fat.

### 2.2. Study Design

From January 2016 through to May 2016, people who were 18 years of age or older and currently pregnant were recruited in-person through three large academic prenatal care centers as well as online through social media. Inclusion criteria were: 18 years of age or older, ability to read/write English, and currently pregnant with a singleton pregnancy. Participants at the health centers were given an information sheet to review regarding the study, and people who elected to participate were then given an informed consent form and a paper copy of a survey packet with a pen and clipboard to complete while they waited for their appointment. Participants who found the study through social media were directed to a Google document, which began with the initial information, and if a person elected to continue, they were then directed to the informed consent and an online survey which was identical to the paper copy completed by participants in the health centers. The survey packet consisted of demographic information, followed by a series of survey instruments. Participants were given a nominal cash incentive of USD 10 for participation.

### 2.3. Instruments

Anti-fat bias was measured using the Discrimination in Medical Settings (DMS) scale. The DMS scale was adapted from existing discrimination scales based on prior studies of healthcare discrimination [31]. It modifies items from the Everyday Discrimination Scale, an instrument that asks about the frequency of experiences with everyday mistreatment [32]. The resulting seven-item instrument assesses responses with a 5-point Likert scale (1—never, 2—rarely, 3—sometimes, 4—most of the time, 5—always). It then asks participants to mark the identities that people felt were at the basis of the discrimination if they had marked ‘3—sometimes’, ‘4—most of the time’, or ‘5—always’ to any of the items. For this analysis, we created a dichotomous variable for anti-fat bias, such that participants were categorized as “yes” if they reported any discrimination related to their weight (2, 3, 4, or 5) in one or more of the situations and “no” if they reported no weight-based discrimination. This approach is consistent with previous studies that utilized dichotomous outcomes of perceived discrimination [33,34].

FOC was measured using the Wijma Delivery Expectancy Questionnaire 10 (WDEQ-10), an adapted version of the WDEQ [35]. Unlike the WDEQ, the WDEQ-10 has demonstrated validity and reliability with diverse samples within the United States [3,36]. The exploratory factor analysis of the WDEQ-10 suggests that there are three different constructs for people experiencing FOC; fear of how they would feel during the birth, fear of death or injury, and fear of how they will be treated by others. We refer to the last construct as Healthcare Fear, measured using two questions related to fear specific to the pregnant person’s healthcare provider and fear of the setting in which they will give birth [36]. For this analysis, we investigated differences in both the total WDEQ-10 score (potentially ranging from 0–60) and the Healthcare Fear score (potentially ranging from 0–12) across categorical variables of interest.

### 2.4. Statistical Analysis

Statistical analysis was performed using IBM SPSS Statistics Version 29.0.2.0 (Armonk, NY, USA). The level of significance was preset to an α of 0.05 (two-tailed). In the original survey, there were seven ethnic and racial categories that people could choose from. These categories were based on the U.S. Census categories with the addition of the category of ‘Middle Eastern’. This category was added in response to the growing critique from the Arab, Middle Eastern, and North African (Arab/MENA) community that being categorized as ‘white’ rejects their identity as an ethnic minority group [37]. Had we conducted this study today, we would have utilized the language of Arab/MENA as opposed to ‘Middle Eastern’ as this is becoming the more commonly used vernacular. Given the small numbers of Middle Eastern, Hispanic/Latina, Asian/Pacific Islander, Native American/Indigenous, and Other respondents, these were collapsed into a single category of Other People of Color. For stratified analytic purposes, we chose to look at comparisons between white and Black participants and exclude the Other category given the small number of people who endorsed these identities.

The DMS scale demonstrated good internal consistency, with the Cronbach alpha coefficient in the current study being 0.84. Similarly, the WDEQ-10 demonstrated good internal consistency, with the Cronbach alpha coefficient in the current study being 0.8. The results of the Kolmogorov–Smirnov test indicated that the WDEQ-10 (D = 0.087, *p* < 0.001) and the Healthcare Fear (D = 0.195, *p* < 0.001) scores significantly deviated from a normal distribution. Therefore, non-parametric tests were used for further analysis.

To examine general differences in the prevalence of anti-fat bias across categories of parity, race, type of provider delivering prenatal care, and trimester in pregnancy, we employed Chi-square (χ^2^) tests.

To investigate potential variations in FOC scores across the two categories of anti-fat bias (“yes” and “no”), we used the non-parametric Mann–Whitney U test. Similarly, to investigate potential variations in FOC scores across the two categories of parity (“nulliparous” and “primip/multiparous”), type of provider delivering prenatal care (“midwife” and “obstetrician”), race (“white” and “Black”), parity (“nulliparous” and “primip/multiparous”), and trimester in pregnancy (“first and second trimester” and “third trimester”), we used Mann–Whitney U tests. We then calculated the effect size of the relationships using Cohen’s d. We then stratified the variables using Mann–Whitney U tests in order to look at differences within the group.

## 3. Results

‘Income’ was excluded from the stratification due to low numbers within each individual group. The levels of missing data were low for the total sample. The missing values appeared to be random but ‘type of provider delivering prenatal care’ was over 5% [38]. All the analyses carried out were based on a complete case analysis. A total of 329 people participated in this study. Our sample was diverse, with more than half of the participants identifying as Black, approximately one-third identifying as White, and the remainder identifying as some other race/ethnicity. All participants were seeing a nurse-midwife or an obstetrician for their prenatal care. Most of the people lived in households with an income of less than USD 30,000/year and had given birth previously (Table 1). Anti-fat bias was reported by more than one-fifth of participants, n = 66 (20.1%). It was the most common discrimination reported by the sample, followed by racial bias, n = 52 (15.8%). Chi-Square tests for independence indicated no significant association between experiencing anti-fat bias, parity, race/ethnicity, and the type of provider delivering prenatal care. However, there was a significant relationship between experiencing anti-fat bias and trimester in pregnancy. People in their first and second trimester of pregnancy were more likely to report anti-fat bias than people in their third trimester.

A Mann–Whitney U test revealed a significant difference in WDEQ-10 scores for people who reported anti-fat bias and people who did not report anti-fat bias. Similarly, another Mann–Whitney U test revealed a significant difference in Healthcare Fear scores for people who reported anti-fat bias and people who did not report anti-fat bias (Table 2).

When the Mann–Whitney U tests were then stratified within the categories, there was a significant association between people experiencing anti-fat bias and increased scores on the WDEQ-10, as well as the scores related specifically to fear of healthcare providers, across all categories (Table 3).

## 4. Discussion

In this study, we examined the association between perceived anti-fat bias from healthcare providers and fear of childbirth (FOC). Pregnant people who reported anti-fat bias from their healthcare provider were significantly more likely to have higher scores on the WDEQ-10, indicating a higher fear of the childbirth experience. Pregnant people who reported anti-fat bias were also significantly more likely to have higher scores related to their perception of how they will be treated by their healthcare providers, also known as Healthcare Fear. Experiencing anti-fat bias from their healthcare providers potentially had a deleterious effect on their anticipation of their birth and their ability to trust that their healthcare providers would keep them safe.

While there was no overall difference by parity, both categories experienced increased overall FOC, as well as increased Healthcare Fear if they experienced anti-fat bias. This was the case for people seeing midwives and people seeing obstetricians, which supports earlier research that midwives are also subject to anti-fat bias.

For people experiencing anti-fat bias, there was a significant racial difference in FOC scores. Black participants were significantly more likely to report overall FOC as well as increased fear related to how they will be treated by providers and in the healthcare system if they experienced anti-fat bias. Anti-fat bias and FOC, particularly among Black patients, are multifaceted issues shaped by historical, cultural, social, and economic factors. Cultural stereotypes and entrenched racism have led to problematic views that associate body size with negative characteristics, intensifying discriminatory attitudes. The historical context of enslavement and systemic oppression continues to influence these societal attitudes that have long linked Black bodies with negative traits.

Fear of healthcare providers can significantly influence how Black patients experience anti-fat bias. This fear often stems from a history of mistreatment, discrimination, and neglect in medical settings. Black patients may avoid seeking medical care due to concerns about being judged or receiving substandard care based on their race and body size. This avoidance can lead to worse health outcomes, reinforcing stereotypes and biases about weight and health. Additionally, fear of providers can create a cycle of mistrust and reduced healthcare engagement, exacerbating health disparities and the effects of anti-fat bias.

Bio-indicators of health aside, healthcare providers frequently categorize fat pregnant people as high risk, which people can experience as punitive, given the cascade of implications of a “high-risk” pregnancy [9]. The relationship between weight and risk during pregnancy is potentially unknown because research exploring fat people’s experiences with pregnancy and birth is rooted in anti-fat bias. The poor pregnancy outcomes reported in the literature have been explained solely by body mass index (BMI), which was never intended to be an individual diagnostic or prescriptive tool [39]. Little or no attention is paid to modifying factors that may increase risk, such as FOC and anti-fat bias.

Implicit and explicit messages from providers about pregnancy risks for fat people frequently result in patients experiencing shame, guilt, and healthcare avoidance, the result of which can have negative implications on health and well-being [40,41]. The high prevalence of anti-fat bias reported by participants in this study indicates that weight discrimination may be one of the most explicit forms of discrimination experienced by pregnant people. This relationship between experiences of anti-fat bias and FOC may be compounded for pregnant people of color who already experience an increased risk for FOC and an earned mistrust of the healthcare system [42].

To our knowledge, this is the first study to explore the association between experiences of anti-fat bias and FOC. For the participants in this study, anti-fat bias from their healthcare provider contributed to FOC. Individually, these two phenomena contribute negatively to pregnancy outcomes and, when experienced together, may substantially increase the risk of negative pregnancy experiences and outcomes.

### Limitations

Our sample size was relatively small, which may impact this study’s ability to fully capture the potential relationships between demographic data and anti-fat bias. While we were able to recruit a majority of non-white participants, there was limited diversity among this sample, outside of participants who identified as Black. Additionally, while the study sample’s income was representative of the cities where data were collected, it was fairly low, thus potentially limiting applicability to populations with higher incomes. Lastly, no biometric data were collected on participants; therefore, there was no way to identify BMI for participants who reported weight discrimination. Therefore, the experience of weight discrimination may not have been grounded in actual body size for participants.

Most studies on fat bias have been conducted in Western countries, including the United States, where cultural norms and values may significantly influence attitudes toward body weight. As such, the findings discussed in this paper are situated within a Western context. We recognize that fat bias may manifest differently in non-Western cultures, and further research is needed to explore these variations.

## 5. Conclusions

It is essential to develop and evaluate training interventions for healthcare providers that target anti-fat bias and provide healthcare providers with recommendations on how to address body health in a way that does not reinforce anti-fat bias but instead builds trust and alliance between pregnant people and their providers. Encouraging positive attitudes, beliefs, skills, and competencies related to caring for fat patients during student training can enhance the readiness of future healthcare professionals and improve the quality of care for fat patients. However, it is essential to go beyond mere awareness and information dissemination. Instead, effective anti-bias training must work to actively develop the skills and competencies of healthcare professionals in recognizing weight bias, as well as making active and deliberate decisions to confront behaviors rooted in anti-fat bias.

Standard implicit bias training has shown little impact on reducing long-established biases [43]. This is largely because they focus on bias as an interpersonal problem, instead of a problem rooted in structural oppressions. Anti-bias training cannot be viewed as a singular opportunity to address bias in the healthcare setting but should be implemented alongside policy changes, political advocacy, environmental shifts in the healthcare setting, and increased access. However, a well-designed anti-bias training program should instead be viewed as a catalyst that produces ripple effects within the healthcare system to promote policy change and political advocacy and challenge systems of oppression. A targeted intervention that provides tangible strategies for healthcare providers to engage in unlearning anti-fat bias on an interpersonal level and to engage in structural change could enhance the quality of care pregnant people receive, increase healthcare utilization, and boost patient satisfaction and trust in the healthcare system. Addressing healthcare fear and distrust necessitates a nuanced approach and requires a collective shift towards more inclusive healthcare spaces that welcome differences around weight and other intersecting identities.

## Figures and Tables

**Table 1:** Participant demographics and prevalence of reported anti-fat bias.

	Anti-Fat Bias (n = 66)	No Anti-Fat Bias (n = 263)	Total (n = 329)	P ^1^
Parity				0.78
Nulliparous	20 (30%)	75 (28%)	95 (28.9%)	
Primip/multiparous	46 (70%)	188 (72%)	234 (71.1%)	
Missing	0	0	0	
Race/ethnicity				0.40
Black	44 (66.7%)	148 (56.3%)	192 (58.4%)	
White	18 (27.3%)	91 (34%)	109 (33.1%)	
Other people of color	4 (6.1%)	11 (4.1%)	15 (4.6%)	
Missing	0	6 (2.2%)	6 (2.7%)	
Type of provider delivering prenatal care				0.57
Midwife	22 (33.3%)	86 (32.7%)	108 (32.8%)	
Obstetrician	38 (57.6%)	125 (47.5%)	163 (49.5%)	
Missing	6 (9.1%)	52 (19.8%)	58 (17.7%)	
Trimester in pregnancy				0.02
First and Second	49 (74.2%)	153 (58.1%)	202 (61.42%)	
Third	17 (25.8%)	106 (40.3%)	123 (37.4%)	
Missing	0	4 (1.5%)	4 (1.2%)	
Income				0.40
<USD 30,000	24 (36.3%)	107 (40.6%)	131 (39.8%)	
USD 30–49,999	9 (13.6%)	48 (18.2%)	57 (17.3%)	
USD 50–99,999	15 (22.7%)	46 (17.4%)	61 (18.5%)	
USD 50–99,999	15 (22.7%)	46 (17.4%)	61 (18.5%)	
Missing	3 (4.5%)	20 (7.6%)	23 (6.9%)	

^1^ Chi-Square tests for independence.

**Table 2:** Associations between FOC and anti-fat bias.

	Anti-Fat Bias (n = 66) Median (25%, 75%, IQR)	No Anti-Fat Bias (n = 261) Median (25%, 75%, IQR)	U	Z	P^2^	Cohen’s d
WDEQ-10	31 (25, 39, 14)	21 (16, 27, 11)	13,686	7.4	<0.001	0.41
Healthcare Fear	7 (5, 9, 4)	3 (2, 5, 3)	14,545	8.65	<0.001	0.48
Missing ^1^		2				

^1^ 2 participants did not complete the WDEQ-10 or Healthcare Fear questions.

**Table 3:** Stratified associations between FOC and Anti-fat bias.

	Nulliparous				Multiparous			
	Anti-fat bias (n = 20) Median (25%, 75%, IQR)	No Anti-fat bias (n = 75) Median (25%, 75%, IQR)	P^2^	d	Anti-fat bias (n = 46) Median (25%, 75%, IQR)	No Anti-fat bias (n = 188) Median (25%, 75%, IQR)	P^2^	d
WDEQ-10	32 (26, 40, 14)	22 (17, 26, 10)	<0.001	0.61	31 (25, 39, 14)	21 (15, 26, 11)	<0.001	0.38
Healthcare Fear	7.5 (5.5, 9, 4)	4 (2, 5, 3)	<0.001	0.73	7 (4, 9, 5)	3 (2, 5, 3)	<0.001	0.46
	Black				White			
	Anti-fat bias (n = 44) Median (25%, 75%, IQR)	No Anti-fat bias (n = 148) Median (25%, 75%, IQR)	P^2^	d	Anti-fat bias (n = 18) Median (25%, 75%, IQR)	No Anti-fat bias (n = 91) Median (25%, 75%, IQR)	P^2^	d
WDEQ-10	37 (28, 43, 15)	21 (16, 26, 10)	<0.001	0.51	27.5 (19, 31, 12)	22 (16.5, 27.5, 12)	0.029	0.21
Healthcare Fear	9 (7.5, 10, 3)	4 (2, 5, 3)	<0.001	0.60	5 (4, 6, 2)	3 (2, 5, 3)	<0.001	0.34
	Midwife				Obstetrician			
	Anti-fat bias (n = 22) Median (25%, 75%, IQR)	No Anti-fat bias (n = 86) Median (25%, 75%, IQR)	P^2^	d	Anti-fat bias (n = 38) Median (25%, 75%, IQR)	No Anti-fat bias (n = 125) Median (25%, 75%, IQR)	P^2^	d
WDEQ-10	30.5 (23, 43, 21)	19 (15, 23, 8)	<0.001	0.40	35.5 (29, 40, 11)	22 (17, 27, 10)	<0.001	0.53
Healthcare Fear	8.5 (4, 10, 6)	3 (2, 4, 2)	<0.001	0.56	8.5 (6, 10, 4)	4 (2, 5, 3)	<0.001	0.58
	First and Second Trimester				Third Trimester			
	Anti-fat bias (n = 49) Median (25%, 75%, IQR)	No Anti-fat bias (n = 153) Median (25%, 75%, IQR)	P^2^	d	Anti-fat bias (n = 17) Median (25%, 75%, IQR)	No Anti-fat bias (n = 106) Median (25%, 75%, IQR)	P^2^	d
WDEQ-10	30 (25, 38, 13)	22 (16, 28, 12)	<0.001	0.37	39 (31, 43, 12)	20 (15, 25, 10)	<0.001	0.53
Healthcare Fear	7 (5, 10, 5)	4 (2, 6, 4)	<0.001	0.49	10 (9, 10, 1)	3 (2, 4, 2)	<0.001	0.56

## Data Availability

The data are available upon request to the corresponding author.
